# Re-creation After Business Failure: A Conceptual Model of the Mediating Role of Psychological Capital

**DOI:** 10.3389/fpsyg.2022.842590

**Published:** 2022-03-02

**Authors:** Roxane De Hoe, Frank Janssen

**Affiliations:** ^1^ICHEC Brussels Management School, Brussels, Belgium; ^2^Louvain Research Institute in Management and Organizations, Université Catholique de Louvain, Louvain-La-Neuve, Belgium

**Keywords:** business failure, learning from failure, psychological capital, conservation of resources model, re-creation, intention to re-create

## Abstract

In case of failure, entrepreneurs could endure various financial, psychological, and social costs. These intertwined costs could affect their learning from failure. All individuals do not react in the same way when dealing with adversity. Rather than focusing on (negative) consequences of business failure, we took a more positive approach by using the Conservation of Resources (COR) model theory to build our conceptual model. Psychological capital, which refers to *“an individual’s positive psychological state of development characterized by high levels of self-efficacy, optimism, hope*, *and resilience,”* could be considered as a resource to recover from entrepreneurial setbacks. We suggest that a high level of psychological capital plays a mediating role in the relationship between the negative consequences of failure and learning from failure. By learning from this experience, failed entrepreneurs will increase their intention to re-create a venture and pursue their entrepreneurial career. This theoretical research, by building a conceptual model based on resources, offers a more positive approach of entrepreneurial failure and investigates key psychological assets, such as psychological capital, that support the development of entrepreneurial resilience rather than the prevention of business failure.

## Introduction

Faced with a complex, uncertain and ever-changing world, the creation and disappearance of firms are common and inherent in the economic process (Cannon and Edmondson, 2005; European Commission, 2007; Ucbasaran et al., 2013). The entrepreneurial journey is paved with plenty of obstacles, possibly leading to failure, certainly in these times of sanitary crisis.

After a long focus on entrepreneurial success, researchers started to investigate the topic of failure by highlighting its importance (DeTienne, 2010; Wennberg et al., 2010; Hessels et al., 2011; Wennberg, 2011; Balcaen et al., 2012; DeTienne and Cardon, 2012; Coad, 2013; DeTienne and Wennberg, 2014; Wennberg and DeTienne, 2014; DeTienne et al., 2015; Justo et al., 2015; Leroy et al., 2015; DeTienne and Wennberg, 2016), starting to change mentalities about business failure and to integrate it as a natural part of the economic cycle. Research is now looking at failure as a way to future success for both entrepreneurs and the economy as a whole (Singh et al., 2007) thanks to the opportunity of learning from it (McGrath, 1999; Minniti and Bygrave, 2001; Shepherd, 2003; Cannon and Edmondson, 2005; Singh et al., 2007; Cope, 2011; Ucbasaran et al., 2013). However, it might also be difficult for an entrepreneur who failed to learn from his/her experience because failure is often seen as an emotionally traumatic event (Shepherd, 2003; Cope, 2011).

In case of failure, entrepreneurs could endure various financial, psychological, and social costs. The financial costs could be associated to a loss of or a reduction in personal income. The social costs are related to the impact of failure on personal and professional relationships, such as divorce for instance (Cope, 2011) and/or loss of an important social network (Harris and Sutton, 1986). The stigma associated to failure is the social devaluation of the person who does not or no longer meet the social norms (Efrat, 2006 cited in Ucbasaran et al., 2013), which is profoundly discrediting (Sutton and Callahan, 1987). Most psychological costs experienced by entrepreneurs after failure are emotional and/or motivational. Negative emotions associated to failure can, for example, be pain, remorse, shame, humiliation, anger, guilt, responsibility, and fear of the unknown (Harris and Sutton, 1986; Shepherd, 2003; Cope, 2011). Concerning the motivational aspects of psychological costs, some authors noted that entrepreneurs who fail have a sense of helplessness that decreases their beliefs in their ability to lead tasks with success in the future and generates rumination that impedes task performance (Bandura, 2001; Shepherd, 2003). Moreover, the intensity of these negative consequences can be influenced by the individual response, as well as by the environmental context in which the entrepreneur finds him/herself (Ucbasaran et al., 2013). The effects and the magnitude of these intertwined costs could affect the process of learning from failure (Ucbasaran et al., 2013).

All individuals do not react to failure in the same way. Some entrepreneurs can re-create a business more easily than others. This could be due to positive emotions. Some researchers believe that the latter play a key role in learning from a business failure and have called to further investigate their role (Byrne and Shepherd, 2013; Ucbasaran et al., 2013). Based on this call, the question at the heart of this conceptual article is: How to explain at the individual level that some entrepreneurs have the intention to re-start after a business failure while others do not?

To answer this question, we focus on the internal resources of the entrepreneurs that could help them to overcome a failure situation, specifically their psychological capital (PsyCap). This concept, coming from the Positive Organizational Behavior and developed by Luthans et al. (2007: 3) refers to *“a positive psychological state of development of the individual characterized by high degrees of self-efficacy, optimism, hope and resilience.”* It could be considered as an asset to recover from entrepreneurial setbacks. Human and social capitals have been studied extensively in the field of entrepreneurship, but internal psychological resources have been left aside. Human capital is related to what a person knows (knowledge, abilities, skills, and experience); whereas social capital refers to people the person knows (his/her relationships and professional networks). According to Luthans and Avolio (2009), PsyCap can be complementary to these two capitals because it concerns who we are and what we become (Luthans et al., 2006b). Therefore, this PsyCap could help entrepreneurs to learn from failure and to make a decision about their subsequent entrepreneurial career.

To build our conceptual model and understand the role of PsyCap to overcome business failure, we use the Conservation of Resources (COR) theory (Hobfoll, 1989, 2002). By being motivated to gain resources, i.e., *“anything perceived by the individual to help attain his or her goals”* (Halbesleben et al., 2014: 1338), individuals try to enrich their resource pool, both to protect them from potential losses and to experience positive well-being. When individuals appraise a situation as a loss of resources, they experiment it as a stressful event. To offset this loss, they mobilize other resources. PsyCap is viewed as a personal characteristic/resource (Gong et al., 2019; Vîrgă et al., 2020) supporting stress resistance. We suggest that a high level of PsyCap plays a mediating role in the relationship between the negative consequences of failure and learning from failure. By learning from this experience, failed entrepreneurs will increase their intention to re-create a venture and pursue their entrepreneurial career. By building a conceptual model based on resources, we offer a more positive approach of entrepreneurial failure and investigate key “psychological assets,” such as PsyCap, that support the development of entrepreneurial resilience rather than the prevention of business failure.

Besides being among the first articles to examine the key role of positive emotions in learning from a business failure and to introduce the concept of PsyCap of in the field of entrepreneurship, our research contributes to the existing literature by being the first one to look at the role of PsyCap in learning from failure and re-creation, as well as to propose a mediating effect of PsyCap on the relationship between business failure consequences and learning from failure.

This article will consist of two parts. First, we will present our theoretical foundations. To this end, we will define entrepreneurial failure, learning from failure and its barriers and facilitators, before explaining the concept of PsyCap, in the light of the COR theory (COR)—which will be the central theory used in this paper—and its links with learning from failure and the intention to re-create. We will illustrate our theoretical argument with our conceptual model. Finally, we will discuss the theoretical and practical implications of this model.

## Theoretical Foundation and Conceptual Framework

This part will consist of five sections. We first define entrepreneurial failure, before explaining learning from failure, its barriers and facilitators. We then present the concept of PsyCap through the light of the COR theory. Finally, we examine the impact of learning from failure and PsyCap on business re-creation.

### Business Failure Definition

Studies on entrepreneurial failure are rather recent. To date, there is no universally accepted definition of entrepreneurial failure. Authors define it based on their own theoretical approach (Smida and Khelil, 2010). The most common definition reduces it to insolvency or bankruptcy (Zacharakis et al., 1999). Even if this definition is useful to operationalize and build samples (Singh et al., 2007), for some authors, entrepreneurial failure cannot be simply be reduced to bankruptcy (McGrath, 1999; Cannon and Edmondson, 2005; Singh et al., 2007; Smida and Khelil, 2010; Ucbasaran et al., 2013).

In addition to the economic aspects, expectations and goals set by the entrepreneur must also be taken into account (McGrath, 1999; Singh et al., 2007; Smida and Khelil, 2010; Ucbasaran et al., 2013). Following recommendation of Shepherd and Patzelt (2017) to use the definition that better suits one’s research question, we endorse the definition of Khelil (2016: 76) who defines entrepreneurial failure as *« a psycho-economic phenomenon characterized by the entry of a new venture into a spiral of insolvency and/or the entrepreneur’s entry into a psychological state of disappointment. In the absence of economic and/or psychological support, entrepreneurs are forced to exit from their entrepreneurial activities »*. It considers the destruction of resources, as well as the entrepreneur’s psychological state. In the absence of a financial and/or moral support, this entrepreneur will see his/her business disappear. This multidimensional and holistic view of failure thus also introduces the concept of “support.” The latter can be both external, through the family, professional, or private network, institutions, etc., and internal, i.e., the own resources of the individual. In this paper, we focus on the entrepreneur’s resources in a failure situation, specifically his/her PsyCap.

### Learning From Failure

Because failure is inherent to the economic life, several researchers in management and entrepreneurship see it as a good opportunity to learn and not repeat the same mistakes (McGrath, 1999; Minniti and Bygrave, 2001; Shepherd, 2003; Cannon and Edmondson, 2005; Cope, 2011; Ucbasaran et al., 2013). Failure can thus contribute to entrepreneurial learning.

Entrepreneurial learning is seen as a dynamic, discontinuous, and changing concept rather than a stable, consistent, and predictable one (Cope, 2005). Indeed, the entrepreneurial process is characterized by significant and critical learning events by which an entrepreneur improves his/her personal and entrepreneurial knowledge that will eventually determine the success of his/her venture (Deakins and Freel, 1998). Entrepreneurs increase their subjective stock of knowledge particularly through non-routine events (Minniti and Bygrave, 2001).

In the context of entrepreneurial learning theory, business failure could be a non-routine event by which an entrepreneur can learn to improve his/her entrepreneurial knowledge and pursue an entrepreneurial career (Shepherd, 2003; Ucbasaran et al., 2010). According to Minniti and Bygrave (2001), both positive and negative experiences shape entrepreneurs’ knowledge and influence the course of their future choices. In line with these authors, Shepherd (2003) defines learning from business failure as the ability for an entrepreneur to revise his/her previous knowledge on how to handle his/her own business efficiently by integrating the feedback information about the reasons why the business failed. From this perspective, failures seem to be “*the seeds of subsequent project success*” (Shepherd et al., 2009a).

Cardon and McGrath (1999 cited in Cope, 2011) stress the importance of considering failure as a “learning journey,” which means that the process of sense-making behind learning from failure is gradual over time and constitutes a dynamic process. This sense-making process is realized through three interconnected mechanisms, i.e., scanning, interpretation, and learning (Gioia and Chittipeddi, 1991; Thomas et al., 1993), the latter acting as a retroaction feedback for scanning and interpreting information (Shepherd et al., 2009a). Specifically, the scanning information is a selective attention to relevant information and a collection of these to promote sensemaking. When information is collected, an individual gathers it into structures appropriate for a better comprehension of its meaning (Taylor and Crocker, 1981; Gioia, 1986). This process refers to the interpretation of the information. Learning dynamics relate to actions taken by an individual (Daft and Weick, 1984) following scanning and interpretation dynamics, leading to significant modifications in one’s current practices (Ginsberg, 1988; Thomas et al., 1993). As mentioned before, these three mechanisms work together because information collected by scanning dynamics is essential for the interpretation (Daft and Weick, 1984). In turn, interpretation structures this information in order to act in a specific way (Gioia and Chittipeddi, 1991), and the action(s) resulting from learning influence, in its(their) turn, scanning and interpretations of new information (Daft and Weick, 1984).

However, learning from entrepreneurial failure is not an easy task. Indeed, negative emotions sometimes interfere with an individual’s attention when he/she is processing information (Mogg et al., 1990), which affects learning (Bower, 1992). Focusing uppermost on emotions that come with failure may interrupt prematurely the information process about potential causes of failure (Bower, 1992). As said before, the magnitude and the intensity of financial, social, and psychological costs may obstruct the learning process of failure (Ucbasaran et al., 2013). The latter is also perceived as intimidating (Rogoff et al., 2004) since the entrepreneur may express a loss of self-esteem (Jenkins et al., 2014), feelings of guilt, shame, and remorse that are difficult to handle (Ucbasaran et al., 2013). Moreover, he/she is not used to deal with it because he/she learned by socialization to keep a distance from negative situations (Cannon and Edmondson, 2005). In this context, learning from failure is not a natural, automatic, or instantaneous act (Wilkinson and Mellahi, 2005). This leads to the first proposition:

*Proposition 1: Financial, psychological and social costs negatively influence learning from failure.*

Some researchers explain how failed entrepreneurs could overcome the negative emotions related to business failure in order to favor learning from failure (Shepherd, 2003; Shepherd et al., 2009a). Through a “grief recovery process,” failed entrepreneurs cope with the loss of his/her business. This process consists of two distinct but complementary strategies: loss and restoration orientations (Shepherd, 2003). These strategies help to manage negative emotions, which contribute in some way to the learning process. This beneficial part depends on both the intensity of the grief (of which symptoms are anger, guilt, anxiety, hopelessness, withdrawal, and depression) and on how far the entrepreneur is in this grief process.

The loss orientation strategy is composed of three phases: a confrontation of loss, a reassessment of the events before, and at the time of failure and the awareness of different causes of failure (Stroebe and Schut, 1999; Shepherd, 2003, 2009). The restoration orientation is entirely different. It consists of distracting from and avoiding all thoughts linked to the loss, as well as, eliminating secondary sources of stress generated by the business failure (Stroebe and Schut, 1999; Shepherd, 2003, 2009). With the oscillation between these dual processes, an entrepreneur adopts the best strategy to handle the loss of his/her business (Shepherd, 2003; Cope, 2011) by regulating his/her emotions. Thereby, emotional interferences are reduced and ability to learn from failure is increased.

An emotion-focused strategy could also help entrepreneurs to manage their negative emotions (Byrne and Shepherd, 2013). Indeed, high negative emotions motivate the entrepreneur to make sense of his/her loss, while higher positive emotions provide him/her with the cognitive resources necessary to facilitate and motivate this sense-making. Cognitive strategies focusing attention on failure and encouraging self-reflection also lead to a better understanding of failure.

These cognitive mechanisms are not the only ones that can minimize the costs of business failure on learning from failure. Other resources such as the PsyCap may have a positive impact and favor the learning process even if failed entrepreneurs experience financial, psychological, and social costs.

### Conservation of Resources Theory and PsyCap

To understand the role of PsyCap in this context, we used the COR theory (Hobfoll, 1989, 2002). The basic tenet of this model of stress is *“that people strive to retain, protect, and build resources and that what is threatening to them is the potential or actual loss of these valued resources”* (Hobfoll, 1989: 516). Three situations explain a stress response: when individuals feel that (1) their resources are threatened, (2) wasted, or (3) their effort to gain new resources is in vain. There is a primacy of resource loss in this theory (Halbesleben et al., 2014) because it is the threat of loss (or the effective loss) of resources that conditions the triggering of stress. For people, losing resources is psychologically more harmful than gaining resources is helpful. Therefore, the second principle of this theory is *“that humans are motivated to protect their current resources and acquire new resources”* (Halbesleben et al., 2014: 1335). This resource investment allows individuals to protect them from resource loss, to bounce back from resource loss, and to acquire new resources.

Resources are anything that individuals perceive as helping to attain their goals (Halbesleben et al., 2014). Hobfoll (1989, 2002) identifies four types of resources: personal characteristics (e.g., self-efficacy, optimism, and well-being), objects resources (e.g., home), conditions (e.g., marital status, tenure, and seniority), and energies (e.g., time, money, and knowledge). The value of these resources is deeply rooted in the sociocultural environment in which an individual lives, which influences his/her reaction and interpretation of the event for his/her well-being. As resources are developed during the life span within cultures that suggest pathways to follow or those not to follow, individuals develop resource caravans. This suggests that when you develop one resource, you can develop others at the same time (Hobfoll, 2002). For this reason, PsyCap is a resource caravan, as will be shown below.

The concept of PsyCap comes from the organizational behavior literature (Luthans et al., 2007). It is viewed as a resource explaining health (Lupsa et al., 2020) or academic performance and reducing burnout and boredom (Vîrgă et al., 2020). In a world where economic uncertainty, constant competition, and perpetual technological advances prevail, companies can gain a sustainable competitive advantage by developing the PsyCap of their human resources (Luthans et al., 2007). In the case of entrepreneurship, an important PsyCap is an asset for the entrepreneur-manager, both for the development of his/her business and in a situation of failure. Despite the limited exploration of PsyCap in the entrepreneurship literature (as mentioned by Welter and Scrimpshire (2021)), several researchers are beginning to investigate its impact on entrepreneurs’ life satisfaction (Bockorny and Youssef-Morgan, 2019), venture performance (Wang et al., 2019), or entrepreneurial intention (Zhao et al., 2020). We study it in the context of entrepreneurial failure because it could help entrepreneurs to recover more easily from the loss of their business and to gain new knowledge by learning from it.

Psychological capital (or PsyCap) is defined by Luthans et al. (2007, p. 3) as:

« *An individual’s positive psychological state of development characterized by:*

1. *Having confidence (self-efficacy) to take on and put in the necessary effort to succeed at challenging tasks;*
2. *Making a positive attribution (optimism) about succeeding now and in the future;*
3. *Persevering toward goals and, when necessary, redirecting paths goals (hope) in order to succeed; and*
4. *When beset by problems and adversity, sustaining and bouncing back and even beyond (resiliency) to attain success* ».

As mentioned in its definition, PsyCap is considered as a state, meaning that individuals can develop these four components to overcome hardships (Luthans, 2002a; Luthans et al., 2006b, 2007; Luthans and Avolio, 2009; Chen and Lim, 2012). This idea that these components can be developed is quite recent. Historically, these four concepts have long been studied as personality traits. For instance, the first theories on resilience considered that an individual was resilient by genes (Coutu, 2002). However, some counter-examples, like resilience from a beloved person’s loss or a traumatic experience, showed that it is more a state (Bonanno, 2004).

It should also be noted that self-efficacy, optimism, hope, and resilience intertwine and interact. Because of their mutual influences, a synergy occurs between them, where the whole is greater than the sum of its parts (Luthans et al., 2006a, 2007). This means that a person who has the will and a clear idea of the path he/she must follow to achieve his/her objectives will be more motivated and capable of overcoming adversity (Luthans et al., 2007). A person who trusts will be able to use hope, optimism, and resilience for specific tasks in different areas of his/her life. A resilient person will be able to use coping mechanisms to develop a realistic and flexible optimism. In turn, self-efficacy, hope, and resilience can also help to develop a positive attribution style to actions under one’s control.

*Self-efficacy* has its origins in the social cognitive theory of Bandura (1986). It refers to *« one’s conviction (or confidence) about his or her abilities to mobilize the motivation, cognitive resources, and courses of action needed to successfully execute a specific task within a given context »* (Stajkovic and Luthans, 1998, p. 66). This sense of efficacy is built through five essential cognitive processes: representation, intention, observation, self-regulation, and self-reflection (Luthans et al., 2007). These cognitive processes allow an individual to take time to reflect on both his/her past successes and failures, learn from them, and use this self-knowledge to progress.

In the field of entrepreneurship, self-efficacy has been widely investigated (Welter and Scrimpshire, 2021). Studies have shown that entrepreneurs have a high degree of self-efficacy (Hayek, 2012). Confident entrepreneurs are motivated to make the necessary effort to successfully conduct their business (Trevelyan, 2011). However, a failure can undermine that trust (Boss and Sims, 2008). In such a situation, it is not general self-efficacy that decreases, but the one related to a specific task (Smith et al., 2006). Therefore, to help an individual to bounce back from a setback, the confidence in his/her ability to succeed the task failed must first restored. From this point of view, self-efficacy could help an entrepreneur to recover from a failure (Boss and Sims 2008). Indeed, by developing it, an individual reflects on his/her past successes and failures, which can contribute to learning.

Inspired by the work of Snyder, *hope* is defined as a positive motivational state based on a sense of achievement from the interaction between the desire (i.e., the energy directed to a goal) and the way/path to get there (i.e., planning to attain goals; Snyder et al., 1991). In other words, hope is «*a cognitive or “thinking” state in which an individual is capable of setting realistic but challenging goals and expectations*» and reaching out to these by his/her self-determination, energy and internal control’s perception (Luthans et al., 2007, p. 66). In addition, hope allows a person to generate alternative ways to achieve his/her desired goals when the original path is not possible.

In the field of entrepreneurship, the perception of internal control, one of the mechanisms that create hope, has been investigated. Research has shown a positive relationship between hope and satisfaction of entrepreneurs owning a business (Jensen and Luthans, 2006; Hayek, 2012). In the case of a failure, nurturing hope could allow the entrepreneur to consider alternative ways to continue an entrepreneurial career if he/she desired it, and therefore could deploy the necessary energy to get there.

Conceptualized by Seligman (1998), *optimism* refers to the attribution of positive events to internal, permanent, and pervasive causes, and negative events to external, temporary, and related to a specific situation cause. In contrast, a pessimistic attribution style interprets positive events as belonging to external and temporary factors related to a particular situation and explain negative events in terms of internal, permanent, and generalized factors. From this point of view, optimistic people tend to consider that the causes of desirable events are under their control (Luthans et al., 2007). Moreover, they expect the causes of these events to persist over time and to be helpful to manage other situations in different areas of their lives. In this way, they see things positively and internalize the positive aspects of their lives, not only in the past and the present, but also in the future. Luthans et al. (2007) suggest that this optimism must be realistic and flexible. It should not be pushed to extremes, in which case an individual could attribute all successes to him/herself, try to control all aspects of his/her life, attribute failures only to external causes, and shirk his/her responsibilities. In this respect, people with a high degree of realistic optimism are capable of gratitude for factors contributing to their success. Similarly, in a situation of failure, they are able to classify information, to establish facts, to learn from their mistakes, to accept what they cannot change, and to move forward.

In the organizational behavior literature, some researchers have shown that the PsyCap optimism can lead to a self-fulfilling prophecy (Peterson and Chang, 2002). In addition, a person with high realistic optimism is both motivating and more motivated to achieve long-term success (Peterson, 2000). Moreover, optimists are more likely to embrace change, to see opportunities in the future, and to focus on these opportunities, even in negative situations (Luthans et al., 2007). Therefore, in a situation of failure, an optimistic entrepreneur would consider this negative situation as a step allowing him to accomplish future success and to identify new entrepreneurial opportunities.

In turn, *resilience* is defined by Luthans (2002a) as the ability to bounce back or quickly recover from a hardship, a conflict, a failure or even positive events such as progress and increased responsibilities. This resilience involves everyday skills and psychological strengths (Masten, 2001; Masten and Reed, 2002). People of all ages and psychological conditions can maintain and nurture resilience. Therefore, resilient people are not exceptional and rare persons (Coutu, 2002).

According to Hayek (2012), resilience is an important characteristic of entrepreneurs because they are known for their determination when they face challenge. But in a situation of failure this resilience can be undermined. Therefore, we suggest that by nurturing or developing their resilience these entrepreneurs can quickly recover from an unsuccessful experience and re-start a business if they wish.

As mentioned above, the four components of PsyCap can also help an entrepreneur to think about what has happened to his/her business. First, we assume that the five cognitive processes (i.e., representation, intentional, observation, self-regulation, and self-reflection) building self-efficacy (Luthans et al., 2007) will help to learn from failure. Hope, optimism, and resilience also are resources that will help to manage failure and its negative consequences to promote learning. These four components act as a resource caravan like as mentioned by the COR theory. Failed entrepreneurs face a loss of resources due to the financial, psychological, and social costs of their business failure. We assume that these costs would be negatively related to PsyCap. However, individuals are motivated to use their resources to face with a loss situation. Drawing on COR theory, and on our review of the literature, we propose that PsyCap would be a positive cognitive resource that buffers the potential threat of a business failure’s consequences on learning from failure. By using their personal resource through PsyCap, failed entrepreneurs gain new resources (energies) such as new knowledge acquired by learning from failure. The mediating role of PsyCap has been highlighted in research on well-being (Sabot and Hicks, 2020), on job performance and job burnout (Gong et al., 2019) as well as on entrepreneurs’ life satisfaction (Bockorny and Youssef-Morgan, 2019), but as far as we know, no research has yet proposed a mediating effect of PsyCap on the relationship between business failure consequences and learning from failure. In this context, we make the following propositions:

*Proposition 2: Financial, psychological and social costs negatively influence PsyCap.*

*Proposition 3: PsyCap mediates the negative impact of financial, psychological and social costs of entrepreneurial failure on learning from failure.*

### Impact of Learning and PsyCap on Re-creating

By learning from his/her entrepreneurial failure, an entrepreneur increases his/her knowledge on different levels: his/her knowledge about him/herself (his/her strengths and weaknesses, skills, abilities, and entrepreneurial approach’s efficacy), the disappearance of his/her venture (strengths and weaknesses of the venture and reasons for failure), the nature of his/her networks and relationships (managing a team, working with a partner, persuading investors, and building valuable collaborations) and the venture management (development of new models of how to manage and grow entrepreneurial ventures; Cope, 2011). These learning outcomes give him/her a future-oriented vision and increase his/her entrepreneurial preparedness’ level to pursue entrepreneurial activities. This new knowledge will be even more useful if the entrepreneur uses it in another business (Shepherd, 2003; Shepherd et al., 2009b), whether it is his/her own new business or if he/she pursues his/her career in the entrepreneurial field without creating a new business (Cope, 2011). This leads to the following proposition:

*Proposition 4: Learning outcomes from failure have a positive influence on the intention of re-creating a new business.*

Other empirical studies also show that people who have started a business are more likely to re-create a new one compared to those who have never tried the entrepreneurial adventure (Caroll and Mosakowski, 1987; Schutjens and Stam, 2006). Entrepreneurial intention is a *sine qua non* condition for entrepreneurial behavior (Krueger, 2003). In their study, Schutjens and Stam (2006) found that most entrepreneurs who have ceased their activity still keep their entrepreneurial intentions at the time of their first business closure. According to these authors, the amount of hours invested in the first company and the experience of running a business contribute to the intention of re-starting a new business.

A few studies have explored the impact of learning from failure on the intention to re-create and the actual re-creation of a new business. Entrepreneurs who have experienced a business exit (by closing, ceasing, or leaving their ventures) have more relevant entrepreneurial skills and identify business opportunities more often than those who did not undergo an entrepreneurial exit (Hessels et al., 2011). In this context, some assume that a new business created by a renascent entrepreneur—that is, an entrepreneur who has exited his/her business and re-enters into entrepreneurship (Stam et al., 2008)—will present better performance (Ucbasaran et al., 2013). A study by Yamakawa et al. (2013) has investigated it empirically by interviewing Japanese entrepreneurs who re-launched a venture after one or more unsuccessful experiences. They studied the influences of cognitive determinants (that is the internal attribution of the cause of failure and intrinsic motivation to re-start a new business) and the experience of failure on the growth of the new business. Entrepreneurs attributing the cause of failure to themselves had better performance when they had had a small number of failures. By contrast, the performance decreased for those who had experienced many failures. For these researchers, entrepreneurial failure is not always beneficial. The relationship between previous failure and the pursuit of an entrepreneurial career is influenced by the cognition of the entrepreneur.

Given that the pursuit of an entrepreneurial career is influenced by entrepreneurs’ cognitions (Yamakawa et al., 2013), the development of PsyCap among failed entrepreneurs should facilitate their learning and promote the re-creation of a new business. PsyCap is a mechanism by which past experiences of failure can shape entrepreneurs to pursue their entrepreneurial journey (Jenkins et al., 2014). By attributing negative events to external, uncontrollable, and varied causes, an individual develops and maintains his/her resilience optimism (Luthans and Youssef, 2004). The latter can help an entrepreneur to preserve his/her entrepreneurial motivations after such a negative experience (Jenkins et al., 2014). The pursuit of an entrepreneurial career could be related to a potential high resilience of the entrepreneur (Jenkins et al., 2014). This leads to the following proposition:

*Proposition 5: PsyCap has a positive influence on the intention to re-create a business.*

In a context of entrepreneurial failure, the development or consolidation of entrepreneurs’ PsyCap can mitigate the negative impact of the failure’s consequences on learning. He/she will learn from his/her mistakes and will therefore be motivated to launch a venture again. This leads to our sixth proposition:

*Proposition 6: If failed entrepreneurs have a high PsyCap, they will learn more easily from their unsuccessful experience, have a more important intention to re-create a business, and will be more likely re-create a new business.*

However, even if an entrepreneur with a high degree of PsyCap manages to learn from failure and wishes to start anew, this willingness can be reduced by too much debt following failure and/or by stigmatization suffered by the entrepreneur in his environment (European Commission, 2007). A good fit between the environment and the individual’s personal attributes shapes the entrepreneurial intentions (Kristof-Brown et al., 2005; Hsu et al., 2017). There are different perceptions of failure among countries or even in several areas in a country (Cardon et al., 2011). Failure is tolerated when the area has a munificent business climate providing support to sustain troubled businesses. In this context, failed entrepreneurs are not stigmatized. In regions where business failure is attributed to a mistake, the stigmatization of entrepreneurs is higher and influences the entrepreneurs’ view of themselves. This stigmatization will affect the entrepreneurs’ willingness to re-create a new business. Moreover, a business failure can generate financial losses. If an entrepreneur has no financial resources on his/her own, he/she will not be able to launch a new business. Hence, financial losses and entrepreneurial stigma would both have a moderating effect on the relationship between the intention to re-create and the actual re-creation following failure (Burchell and Hughes, 2006; Stam et al., 2008; Cope, 2011; Simmons et al., 2014). This leads us to the following proposition:

*Proposition 7: Debt and/or stigma moderate the relationship between intention to re-create and actual re-creation. More specifically, the more debt an entrepreneur has and/or the more stigmatized he/she is after failure, the less positive the relationship between the intention to re-create and the actual re-creation will be.*

### Conceptual Framework

**Figure 1:**
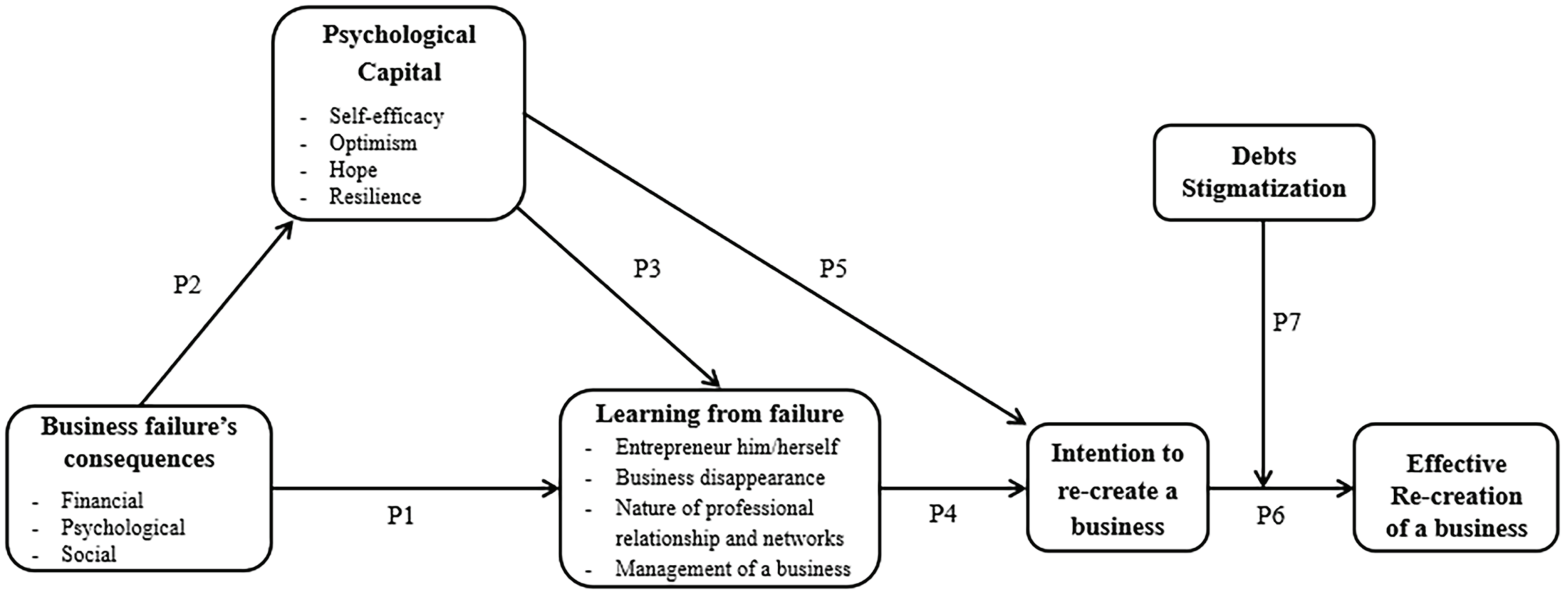
Conceptual model of the impact of psychological capital (PsyCap) on learning from failure and intention to re-create.

Figure 1 presents our conceptual model. It suggests that PsyCap would promote (1) learning in the context of entrepreneurial failure and (2) the intention of the failed entrepreneur to pursue his/her entrepreneurial career. The failure’s consequences will prevent the good process of learning from failure (Ucbasaran et al., 2013; P1). We propose that there is a negative relation between business failure consequences and PsyCap (P2). However, in a situation of loss of resources, such as business failure, a failed entrepreneur will be motivated to gain new resources through learning from failure. To acquire these new resources, he/she will use other resources he/she has, such as his/her PsyCap. If this positive state encourages the entrepreneur to learn from his/her mistakes (P3), he/she could manifest the desire to continue his/her entrepreneurial career by not repeating the same mistakes (P4 and P5). Since the intention to create is a *sine qua non* condition to the effective creation (Krueger, 2003), we assume that the intention of re-creating a new business will help to lead to the real re-creation of a new venture (P6). However, this effective recreation could be reduced if the entrepreneur has too much debt and/or feels strongly stigmatized by his environment, even if he has a high PsyCap and feels he has learned from his failure (P7).

## Discussion

Many researchers agree that entrepreneurs learn from their entrepreneurial failure (McGrath, 1999; Minniti and Bygrave, 2001; Shepherd, 2003; Cannon and Edmondson, 2005; Cope, 2011; Ucbasaran et al., 2013). However, given the financial, psychological, and social consequences of business failure and of the social stigmatization of these entrepreneurs, this learning is not an easy task (Ucbasaran et al., 2013). Despite these barriers, 3%–8% of them still re-start a new activity. How to explain this figure? Our conceptual model can be of some help. Its originality stems from a positive approach to promote learning from failure through PsyCap and their impact on the intention to re-create, as well as the effective re-creation of a subsequent business. Drawing on COR theory (Hobfoll, 1989, 2002), we considered that the PsyCap is a personal resource that failed entrepreneurs can mobilize to gain new knowledge about entrepreneurship. We suggest that a high level of PsyCap buffers the negative effects of the failure’s consequences on entrepreneurial learning process. In addition, we assume that it also has a positive effect on the intention to re-create a business; this intention can be followed by effective re-creation of a new venture. Similarly, the accumulation of knowledge on the entrepreneur him/herself, the ending of his/her business, and the management of a business and professional relationships can positively influence his/her intention to create a new venture and really do it.

Understanding the barriers and facilitators of learning from failure and their implications for the pursuit of an entrepreneurial career process from a theoretical point of view offers interesting avenues for future research.

If some authors have begun to investigate the cognitive and emotional processes that influence the performance of a new business after previous business failures (Yamakawa et al., 2013) or to make sense of this failure (Byrne and Shepherd, 2013), few studies have focused on mechanisms that facilitate learning from entrepreneurial failure. Through our theoretical model, we try to answer the following question: Does a positive psychological state allow better learning and re-starting after a business failure? The positive approach of PsyCap would enable entrepreneurs to capitalize on their failure’s experience in order to better bounce back from it. A study on job search has shown that through interventions and training to help job seekers to develop their PsyCap, they increased their perceived employability in their job search (Chen and Lim, 2012). By developing a high degree of PsyCap, unemployed people are more confident about their abilities and skills, are more optimistic about the future, do not give up their job search, and invent solutions to overcome obstacles in their job search. This positive attitude encourages them to look for opportunities rather than to attribute their difficulties to external causes and blame themselves (Chen and Lim, 2012). While a job search situation is not similar to an entrepreneurial failure, we believe that the development of PsyCap may have similar effects on the ability of entrepreneurs to learn from their failure and continue their entrepreneurial career.

The concept of PsyCap is used more and more in the field of organizational behavior. Some authors have also started to introduce it in the field of entrepreneurship (Jensen and Luthans, 2006; Jenkins et al., 2014). Its application to the entrepreneurial process could be considered at the venture creation stage, as a determinant of opportunities identification or intention to create, or later, as a determinant of performance. Welter and Scrimpshire (2021) have recently called to incorporate PsyCap into entrepreneurship research and develop research questions on topics such as opportunity evaluation or the process of entrepreneurship.

Besides PsyCap, other cognitive or affective mechanisms may also be relevant to promote learning from failure in order to re-create a new business. In particular, we believe in the potential of the concept of hardiness developed by Maddi (2013). Luthans et al. (2007) also suggest that cognitive processes, such as creativity and wisdom, as well as emotional processes, perceived well-being, flow (that is a state of maximum concentration) and humor should be investigated, as these could also be constitutive of PsyCap. Based on the COR theory, these other mechanisms are resources forming a resource caravan. The more resources an individual has, the more he/she will be prone to gain additional resources and recover from losses (Halbesleben et al., 2014).

In terms of the practical implications, PsyCap is a tool that can be exploited for the development of the entrepreneur in general and, more specifically, of the entrepreneur in a business failure context. This concept has two major advantages: it can be developed and is available to everyone (Luthans, 2002a,b; Fleig-Palmer et al., 2009; Chen and Lim, 2012). Providing training for entrepreneurs to develop their PsyCap would be in line with the recommendations of the European Commission. Indeed, in its 2007 report, it recommended to develop psychological and technical supports for entrepreneurs who have failed, through, for instance, training and specific supervision. Considering that the half of newly established firms does not survive the first 5 years after creation (European Commission, 2007) and that very few failed entrepreneurs re-launch a new business thereafter, it would seem appropriate to focus on these entrepreneurs.

Finally, we believe that PsyCap has a bright future in entrepreneurship research at large. In addition to its usefulness in understanding learning from failure and re-creation, it could be investigated in the creation process and/or in opportunities identification or as a factor promoting the leadership of the entrepreneur and his/her business’ performance.

## Author Contributions

RD and FJ contributed to formulating the conceptual framework, analyzing the literature, and writing and editing the manuscript. All authors contributed to the article and approved the submitted version.

## Conflict of Interest

The authors declare that the research was conducted in the absence of any commercial or financial relationships that could be construed as a potential conflict of interest.

## Publisher’s Note

All claims expressed in this article are solely those of the authors and do not necessarily represent those of their affiliated organizations, or those of the publisher, the editors and the reviewers. Any product that may be evaluated in this article, or claim that may be made by its manufacturer, is not guaranteed or endorsed by the publisher.

## References

[ref1] Balcaen S. Manigart S. Buyze J. Ooghe H. (2012). Firm exit after distress: differentiating between bankruptcy, voluntary liquidation and M&A. Small Bus. Econ. 39, 949–975. doi: 10.1007/s11187-011-9342-7

[ref2] Bandura A. (1986). Social Foundations of Thought and Action: A Social Cognitive Theory. Englewood Cliffs, NJ, Prentice-Hall: Cambridge University Press, 617.

[ref100] Bandura A. (2001). Social cognitive theory: an agentic perspective. Annu. Rev. Psychol. 52, 1–26. doi: 10.1146/annurev.psych.52.1.1, PMID: 11148297

[ref3] Bockorny K. Youssef-Morgan C. M. (2019). Entrepreneurs’ courage, psychological capital, and life satisfaction. Front. Psychol. 10:789. doi: 10.3389/fpsyg.2019.00789, PMID: 31024410 PMC6461011

[ref4] Bonanno G. A. (2004). Loss, trauma, and human resilience: have we underestimated the human capacity to thrive after extremely aversive events? Am. Psychol. 59, 20–28. doi: 10.1037/0003-066X.59.1.20, PMID: 14736317

[ref5] Boss A. D. Sims H. P. Jr. (2008). Everyone fails! Using emotion regulation and self-leadership for recovery. J. Manag. Psychol. 23, 135–150. doi: 10.1108/02683940810850781

[ref6] Bower G. H. (1992). “How might emotions affect learning?” in The Handbook of Emotion and Memory: Research and Theory. ed. Christianson S. (Hillsdale, NJ, England: Lawrence Erlbaum), 3–31.

[ref7] Burchell B. Hughes A. (2006). The Stigma of Failure: An International Comparison of Failure Tolerance and Second Chancing. Working paper. England, University of Cambridge, p. 38.

[ref8] Byrne O. Shepherd D. A. (2013). Different strokes for different folks: entrepreneurial narratives of emotion, cognition, and making sense of business failure. Entrep. Theory Pract. 39:12046. doi: 10.1111/etap.12046

[ref9] Cannon M. D. Edmondson A. C. (2005). Failing to learn and learning to fail (intelligently). Long Range Plan. 38, 299–319. doi: 10.1016/j.lrp.2005.04.005

[ref10] Cardon M. S. McGrath R. G. (1999). “When the Going Gets Tough. Toward a Psychology of Entrepreneurial Failure and Re-Motivation,” in Frontiers of Entrepreneurship Research. Babson College.

[ref11] Cardon M. S. Stevens C. E. Potter D. R. (2011). Misfortunes or mistakes? Cultural sensemaking of entrepreneurial failure. J. Bus. Ventur. 26, 79–92. doi: 10.1016/j.jbusvent.2009.06.004, PMID: 35025600

[ref12] Caroll G. R. Mosakowski E. (1987). The career dynamics of self-employment. Adm. Sci. Q. 32, 570–589. doi: 10.2307/2392884

[ref13] Chen D. J. Q. Lim V. K. G. (2012). Strength in adversity: the influence of psychological capital on job search. J. Organ. Behav. 33, 811–839. doi: 10.1002/job.1814

[ref14] Coad A. (2013). Death is not a success: reflections on business exit. Int. Small Bus. J. 32, 721–732. doi: 10.1177/0266242612475104

[ref15] Cope J. (2005). Toward a dynamic learning perspective of entrepreneurship. Entrep. Theory Pract. 29, 373–397. doi: 10.1111/j.1540-6520.2005.00090.x

[ref16] Cope J. (2011). Entrepreneurial learning from failure: an interpretative phenomenological analysis. J. Bus. Ventur. 26, 604–623. doi: 10.1016/j.jbusvent.2010.06.002

[ref17] Coutu D. L. (2002). How resilience works. Harv. Bus. Rev. 80, 46–55. PMID: 12024758

[ref18] Daft R. L. Weick K. E. (1984). Toward a model of organizations as interpretation systems. Acad. Manag. Rev. 9, 284–295. doi: 10.2307/258441

[ref19] Deakins D. Freel M. (1998). Entrepreneurial learning and the growth process in SMEs. Learn. Organ. 5, 144–155. doi: 10.1108/09696479810223428

[ref20] DeTienne D. R. (2010). Entrepreneurial exit as a critical component of the entrepreneurial process: theoretical development. J. Bus. Ventur. 25, 203–215. doi: 10.1016/j.jbusvent.2008.05.004

[ref21] DeTienne D. R. Cardon M. S. (2012). Impact of founder experience on exit intentions. Small Bus. Econ. 38, 351–374. doi: 10.1007/s11187-010-9284-5

[ref22] DeTienne D. R. McKelvie A. Chandler G. N. (2015). Making sense of entrepreneurial exit strategies: a typology and test. J. Bus. Ventur. 30, 255–272. doi: 10.1016/j.jbusvent.2014.07.007

[ref23] DeTienne D. R. Wennberg K. (2014). “Small business exit: review of past research, theoretical considerations and suggestions for future research,” in Small Businesses in a Global Economy: Creating and Managing Successful Organizations. ed. Newbert S. (Westport, CT: Praeger)

[ref24] DeTienne D. Wennberg K. (2016). Studying exit from entrepreneurship: new directions and insights. Int. Small Bus. J. 34, 151–156. doi: 10.1177/0266242615601202

[ref200] Efrat R. (2006). Bankruptcy stigma: Plausible cases for shifting norms. Emory Bankruptcy Developments Journal 22, 481–520.

[ref26] European Commission (2007). Overcoming the Stigma of Business Failure—For a Second Chance Policy, Bruxelles

[ref28] Fleig-Palmer M. M. Luthans K. W. Mandernach B. J. (2009). Successful reemployment Through resiliency development. J. Career Dev. 35, 228–247. doi: 10.1177/0894845308327271

[ref29] Ginsberg A. (1988). Measuring and modelling changes in strategy: theoretical foundations and emperical directions. Strateg. Manag. J. 9, 559–575. doi: 10.1002/smj.4250090604

[ref30] Gioia D. A. (1986). “Symbols, scripts, and sense making: creating meaning in the organizational experience,” in The thinking organization. eds. Sims H. P. J. Gioia D. A. (San Francisco, California: Jossey-Bass), 49–74.

[ref31] Gioia D. A. Chittipeddi K. (1991). Sensemaking and sensegiving in strategic change initiation. Strateg. Manag. J. 12, 433–448. doi: 10.1002/smj.4250120604

[ref32] Gong Z. Chen Y. Wang Y. (2019). The influence of emotional intelligence on job burnout and job performance: mediating effect of psychological capital. Front. Psychol. 10:2707. doi: 10.3389/fpsyg.2019.02707, PMID: 31920783 PMC6916327

[ref33] Halbesleben J. Neveu J.-P. Paustian-Underdahl S. Westman M. (2014). Getting to the “COR”: understanding the role of resources in conservation of resources theory. J. Manag. 40, 1334–1364. doi: 10.1177/0149206314527130

[ref34] Harris S. G. Sutton R. I. (1986). Functions of parting ceremonies in dying organizations. Acad. Manag. J. 29, 5–30.

[ref35] Hayek M. (2012). Control beliefs and positive PsyCap. Can nascent entrepreneurs discriminate between what can and cannot be controlled? J. Manag. Res. 12, 3–13.

[ref36] Hessels J. Grilo I. Thurik R. Zwan P. (2011). Entrepreneurial exit and entrepreneurial engagement. J. Evol. Econ. 21, 447–471. doi: 10.1007/s00191-010-0190-4

[ref37] Hobfoll S. E. (1989). Conservation of resources: A new attempt at conceptualizing stress. Am. Psychol. 44, 513–524. doi: 10.1037/0003-066X.44.3.513, PMID: 2648906

[ref38] Hobfoll S. E. (2002). Social and psychological resources and adaptation. Rev. Gen. Psychol. 6, 307–324. doi: 10.1037/1089-2680.6.4.307, PMID: 35096285

[ref39] Hsu D. K. Shinnar R. S. Powell B. C. Betty C. S. (2017). Intentions to reenter venture creation: the effect of entrepreneurial experience and organizational climate. Int. Small Bus. J. 35, 928–948. doi: 10.1177/0266242616686646

[ref40] Jenkins A. S. Wiklund J. Brundin E. (2014). Individual responses to firm failure: appraisals, grief, and the influence of prior failure experience. J. Bus. Ventur. 29, 17–33. doi: 10.1016/j.jbusvent.2012.10.006

[ref41] Jensen S. M. Luthans F. (2006). Relationship between Entrepreneurs' psychological capital and their authentic leadership. J. Manag. Issues 18, 254–273.

[ref42] Justo R. DeTienne D. R. Sieger P. (2015). Failure or voluntary exit? Reassessing the female underperformance hypothesis. J. Bus. Ventur. 30, 775–792. doi: 10.1016/j.jbusvent.2015.04.004, PMID: 35148570

[ref43] Kristof-Brown A. L. Zimmerman R. D. Johnson E. C. (2005). Consequences of individuals’ fit at work: a meta-analysis of person-job, person-organization, person-group, and person-supervisor fit. Pers. Psychol. 58, 281–342. doi: 10.1111/j.1744-6570.2005.00672.x

[ref44] Krueger N. (2003). “The cognitive psychology of entrepreneurship,” in Handbook of Entrepreneurship Research. An Interdisciplinarity Introduction. eds. Acs Z. Audretsch D. (Boston: Kluwer), 105–140.

[ref45] Leroy H. Manigart S. Meuleman M. Collewaert V. (2015). Understanding the continuation of firm activities when entrepreneurs exit their firms: using theory of planned behavior. J. Small Bus. Manag. 53, 400–415. doi: 10.1111/jsbm.12077

[ref46] Lupsa D. Baciu L. Vîrgă D. (2020). Psychological capital, oragnizational justice and health: the mediating role of work engagement. Pers. Rev. 49, 87–103. doi: 10.1108/PR-08-2018-0292

[ref47] Luthans F. (2002a). The need for and meaning of positive organizational behavior. J. Organ. Behav. 23, 695–706. doi: 10.1002/job.165, PMID: 33865755

[ref48] Luthans F. (2002b). Positive organizational behavior: developing and managing psychological strengths. Acad. Manag. Exec. 16, 57–72. doi: 10.5465/AME.2002.6640181

[ref49] Luthans F. Avey J. B. Avolio B. J. Norman S. M. Combs G. M. (2006a). Psychological capital development: toward a micro-intervention. J. Organ. Behav. 27, 387–393. doi: 10.1002/job.373

[ref50] Luthans F. Avolio B. J. (2009). The “point” of positive organizational behavior. J. Organ. Behav. 30, 291–307. doi: 10.1002/job.589, PMID: 35143498

[ref51] Luthans F. Vogelgesang G. R. Lester P. B. (2006b). Developing the psychological capital of resiliency. Hum. Resour. Dev. Rev. 5, 25–44. doi: 10.1177/1534484305285335, PMID: 21631886

[ref300] Luthans F. Youssef C. M. (2004). Human, social, and now positive psychological capital management. Occup. Environ. Med. 33, 143–160. doi: 10.1136/oemed-2016-103726

[ref52] Luthans F. Youssef C. M. Avolio B. J. (2007). Psychological Capital: Developing the Human Competitive Edge. New York: Oxford University Press, 246.

[ref53] Maddi S. (2013). Hardiness: Turning Stressful Circumstances into Resilient Growth. Germany: Springer, 88

[ref54] Masten A. (2001). Ordinary magic: resilience process in development. Am. Psychol. 56, 227–238. doi: 10.1037/0003-066X.56.3.227, PMID: 11315249

[ref55] Masten A. Reed M. (2002). “Resilience in development,” in Handbook of Positive Psychology. eds. Snyder C. Lopez S. (Oxford, UK: Oxford University Pres), 74–88.

[ref56] McGrath R. G. (1999). Falling forward: real options reasoning and entrepreneurial failure. Acad. Manag. Rev. 24, 13–30. doi: 10.5465/amr.1999.1580438

[ref57] Minniti M. Bygrave W. (2001). A dynamic model of entrepreneurial learning. Entrep. Theory Pract. 25, 5–16. doi: 10.1177/104225870102500301, PMID: 34616339

[ref58] Mogg K. Mathews A. Bird C. MacGregor-Morris R. (1990). Effects of stress and anxiety on the processing of threat stimuli. J. Pers. Soc. Psychol. 59, 1230–1237. doi: 10.1037/0022-3514.59.6.1230, PMID: 2283589

[ref59] Peterson C. (2000). The future of optimism. Am. Psychol. 55, 44–55. doi: 10.1037/0003-066X.55.1.44, PMID: 11392864

[ref60] Peterson C. Chang E. (2002). “Optimism and flourishing,” in Flourishing: Positive Psychology and the Life Well-Lived. eds. Keyes C. Haidt J. (Washington DC: American Psychological Association), 55–79.

[ref61] Rogoff E. G. Lee M. S. Suh D. C. (2004). "Who do it?"—Attributions by entrepreneurs and experts of the factors that cause and impede small business success. J. Small Bus. Manag. 42, 364–376. doi: 10.1111/j.1540-627X.2004.00117.x

[ref62] Sabot D. L. Hicks R. E. (2020). Does psychological capital mediate the impact of dysfunctional sleep beliefs on well-being? Heliyon 6:e04314. doi: 10.1016/j.heliyon.2020.e04314, PMID: 32617422 PMC7322688

[ref63] Schutjens V. Stam E. (2006). Starting Anew: Entrepreneurial Intentions and Realizations Susbsequent to Business Closure. The papers on Entrepreneurship, Growth and Public Policy, Vol. ERS-2006-015-ORG: 23, Jena, Germany, Max Planck Institute of Economics.

[ref64] Seligman M. (1998). Learned Optimism. New York: Pocket Books. 336

[ref400] Simmons S. A. Wiklund J. Levie J. (2014). Stigma and business failure: implications for entrepreneurs’ career choices. Small Business Economics. 42, 485–505.

[ref65] Shepherd D. A. (2003). Learning from business failure: propositions of grief recovery for the self-employed. Acad. Manag. Rev. 28, 318–328. doi: 10.2307/30040715

[ref66] Shepherd D. A. (2009). Grief recovery from the loss of a family business: a multi- and meso-level theory. J. Bus. Ventur. 24, 81–97. doi: 10.1016/j.jbusvent.2007.09.003

[ref67] Shepherd D. A. Covin J. G. Kuratko D. F. (2009a). Project failure from corporate entrepreneurship: managing the grief process. J. Bus. Ventur. 24, 588–600. doi: 10.1016/j.jbusvent.2008.01.009

[ref68] Shepherd D. A. Wiklund J. Haynie J. M. (2009b). Moving forward: balancing the financial and emotional costs of business failure. J. Bus. Ventur. 24, 134–148. doi: 10.1016/j.jbusvent.2007.10.002

[ref69] Singh S. Corner P. Pavlovich K. (2007). Coping with entrepreneurial failure. J. Manag. Organ. 13, 331–344. doi: 10.5172/jmo.2007.13.4.331, PMID: 34209218

[ref500] Smida A. Khelil N. (2010). Repenser l’échec entrepreneurial des petites entreprises émergentes. Proposition d’une typologie s’appuyant sur une approche intégrative. Revue Internationale P.M.E. : economie et gestion de la petite et moyenne entreprise. 23, 65–106.

[ref70] Smith S. A. Kass S. J. Rotunda R. J. Schneider S. K. (2006). If at first you don’t succeed: effects of failure on general and task-specific self-efficacy and performance. N. Am. J. Psychol. 8, 171–182.

[ref71] Snyder C. Irving L. Anderson J. (1991). “Hope and health: measuring the will and the ways,” in Handbook of Social and Clinical Psychology. eds. Snyder C. Forsyth D. (Elmsford, NY: Pergamon), 285–305.

[ref72] Stajkovic A. Luthans F. (1998). Social cognitive theory and self-efficacy: going beyond traditional motivational and behavioral approaches. Organ. Dyn. 26, 62–74. doi: 10.1016/S0090-2616(98)90006-7

[ref73] Stam E. Audretsch D. Meijaard J. (2008). Renascent entrepreneurship. J. Evol. Econ. 18, 493–507. doi: 10.1007/s00191-008-0095-7

[ref74] Stroebe M. Schut H. (1999). The dual process model of coping with bereavement: rational and description. Death Stud. 23, 197–224. doi: 10.1080/074811899201046, PMID: 10848151

[ref75] Sutton R. I. Callahan A. L. (1987). The stigma of bankuptcy: spoiled organizational image and its management. Acad. Manag. J. 30, 405–436.

[ref76] Taylor S. E. Crocker J. (1981). “Schematic bases of social information processing” in Social cognition. eds. Higgins E. T. Herman C. P. Zanna M. P. Vol. 1 (Hillsdale, NJ: Lawrence Erlbaum), 89–133.

[ref77] Thomas J. B. Clark S. M. Gioia D. A. (1993). Strategic sensemaking and organizational performance: linkages among scanning, interpretation, action, and outcomes. Acad. Manag. J. 36, 239–270. PMID: 10125120

[ref78] Trevelyan R. (2011). Self-efficacy and effort in new venture development. J. Manag. Organ. 17, 2–16. doi: 10.5172/jmo.2011.17.1.2

[ref79] Ucbasaran D. Shepherd D. A. Lockett A. Lyon S. J. (2013). Life after business failure: the process and consequences of business failure for entrepreneurs. J. Manag. 39, 163–202. doi: 10.1177/0149206312457823

[ref80] Ucbasaran D. Westhead P. Wright M. Flores M. (2010). The nature of entrepreneurial experience, business failure and comparative optimism. J. Bus. Ventur. 25, 541–555. doi: 10.1016/j.jbusvent.2009.04.001

[ref81] Vîrgă D. Pattusamy M. Kumar D. P. (2020). How psychological capital is related to academic performance, burnout, and boredom? The mediating role of study engagement. Curr. Psychol. doi: 10.1007/s12144-020-01162-9

[ref82] Wang Y. Tsai C.-H. Lin D. D. Enkhbuyant O. Cai J. (2019). Effects of human, relational, and psychological capitals on new venture performance. Front. Psychol. 10:1071. doi: 10.3389/fpsyg.2019.01071, PMID: 31275186 PMC6591455

[ref83] Welter C. Scrimpshire A. (2021). The missing capital: the case for psychological capital in entrepreneurship research. J. Bus. Ventur. Insights 16:e00267. doi: 10.1016/j.jbvi.2021.e00267, PMID: 31507268

[ref84] Wennberg K. (2011). “Entrepreneurial exit,” in World Encyklopedia of Entrepreneurship. ed. Dana L. P. (Cheltenham, UK: Edward Elgar), 170–177.

[ref85] Wennberg K. DeTienne D. R. (2014). What do we really mean when we talk about 'exit'? A critical review of research on entrepreneurial exit. Int. Small Bus. J. 32, 4–16. doi: 10.1177/0266242613517126

[ref86] Wennberg K. Wiklund J. DeTienne D. R. Cardon M. S. (2010). Reconceptualizing entrepreneurial exit: divergent exit routes and their drivers. J. Bus. Ventur. 25, 361–375. doi: 10.1016/j.jbusvent.2009.01.001

[ref87] Wilkinson A. Mellahi K. (2005). Organizational failure. Long Range Plan. 38, 233–238. doi: 10.1016/j.lrp.2005.05.009, PMID: 35142861

[ref88] Yamakawa Y. Peng M. Deeds D. (2013). Rising from the ashes: cognitive determinants of venture growth after entrepreneurial failure. Entrep. Theory Pract. 39:12047. doi: 10.1111/etap.12047

[ref89] Zacharakis A. Meyer G. DeCastro J. (1999). Differing perceptions of new venture failure: a matched exploratory study of venture capitalists and entrepreneurs. J. Small Bus. Manag. 37, 1–14.

[ref90] Zhao J. Wei G. Chen K.-H. Yien J.-M. (2020). Psychological capital and university students’ entrepreneurial intention in China: mediation effect of entrepreneurial capitals. Front. Psychol. 10:2984. doi: 10.3389/fpsyg.2019.02984, PMID: 32038375 PMC6989491

